# Sequence Polymorphism and Expression Variability of *Crassostrea* *gigas* Immune Related Genes Discriminate Two Oyster Lines Contrasted in Term of Resistance to Summer Mortalities

**DOI:** 10.1371/journal.pone.0075900

**Published:** 2013-09-26

**Authors:** Paulina Schmitt, Adrien Santini, Agnès Vergnes, Lionel Degremont, Julien de Lorgeril

**Affiliations:** 1 Institut Français de Recherche pour l’Exploitation de la Mer, Centre National de la Recherche Scientifique, Université de Montpellier 2, Université de Montpellier 1, Institut de la Recherche pour le Développement, UMR 5119 "Ecologie des Systèmes Marins Côtiers", Montpellier, France; 2 Institut Français de Recherche pour l’Exploitation de la Mer, Laboratoire de Génétique et de Pathologie des Mollusques Marins, La Tremblade, France; The Ohio State University, United States of America

## Abstract

Summer mortalities of *Crassostrea* *gigas* are a major concern in oyster aquaculture. They are the result of a complex interaction between the host, pathogens and environmental factors. Oyster genetics have been identified as an essential determinant of oyster susceptibility to summer mortalities. As the capability of oysters to circumvent diseases depends in part on their immune defenses, we aimed to analyze the gene expression and sequence polymorphism of 42 immune related genes in two oyster lines selected for their “High” (H) and “Low” (L) survival to summer mortalities. Results showed that the variability of gene expression and the sequence polymorphism acting on particular genes could enable the discrimination between H and L oyster lines. Besides, a higher sequence polymorphism was observed on the L line affecting 11 of the 42 analyzed genes. By analyzing gene expression, sequence polymorphism and gene copy number of two antimicrobial peptide families (*Cg-Defs* and *Cg-Prp*), and an antimicrobial protein (*Cg-BPI*) on individual oysters, we showed that gene expression and/or sequence polymorphism could also discriminate H and L oyster lines. Finally, we observed a positive correlation between the gene expression and the gene copy number of antimicrobials and that sequence polymorphism could be encoded in the genome. Overall, this study gives new insights in the relationship between oyster immunity and divergent phenotypes, and discusses the potential implication of antimicrobial diversity in oyster survival to summer mortalities.

## Introduction

The Pacific oyster *Crassostrea* *gigas* is an important aquaculture species, which is farmed worldwide. In their marine environment, oysters are in permanent contact with pathogenic and opportunistic pathogens. For several decades, high levels of mortalities have occurred in farmed *C* *. gigas* during the summer months in many countries and they are a major concern to oyster aquaculture [1]. These mortalities result from a complex interaction of multiple factors, including the environment, oyster genetics and physiology, and the presence of several infective agents, including an Oyster Herpes virus and bacteria of the *Vibrio* genus, particularly *V. splendidus* and *V* *. aestuarianus* strains [2–4].

In the context of “summer mortalities” from 2001 to 2006, several studies identified a strong genetic basis and a positive response to selection to increase or decrease survival in juvenile oysters. Results from these studies suggested that selective breeding could efficiently improve oyster survival [5–7]. Oyster lines selected for high (H) and low (L) resistance to summer mortalities were generated and show differential survival capacity in the context of the massive mortality reported in juvenile *C* *. gigas* in France since 2008 [8]. The H line was resistant to the Ostreid Herpes virus type 1 associated to summer mortalities while the L line was highly susceptible to the disease. Interestingly, the relationship between immunity and resistant lines has been recently highlighted by the over representation of immune signaling genes in a H survival oyster line compared to a L survival line during an summer mortality event [9]. In addition, reproduction allocation and antioxidant defenses were shown to differ between H and L oyster lines in response to summer mortality events [10].

Because it has been postulated that the capability of oysters to circumvent diseases depends on their defense system, many research efforts have been made over the past years to characterize the molecular processes implicated in their defense reactions. Several families of antimicrobial peptides (AMP) and protein have been characterized from oyster hemocytes and various epithelia. Members of the defensin family (*Cg*-Defs) have been identified from the oyster mantle (*Cg*-Defm) [11] and hemocytes (*Cg*-Defh1 and *Cg*-Defh2) [12], both being constitutively expressed in different tissues and encoded by different genes. A proline-rich AMP called *Cg*-Prp was also identified from hemocytes [13]. Later, a shorter *Cg-Prp* variant, produced by an indel of six nucleotides in the cationic domain coding sequence was described, being encoded by a different gene [14]. Both variants display antimicrobial activities in synergy with oyster defensins [15]. In addition, a bactericidal/permeability-increasing protein (*Cg*-BPI) was identified in oyster hemocytes and epithelia. The gene expression of *Cg*-BPI was shown to be inducible in hemocytes of immune challenged oysters and constitutive in several epithelia [16]. Besides, two variants from the Big defensin family (*Cg-BigDef1* and *Cg-BigDef2*) have been identified and described as inducible AMPs in hemocytes by bacterial challenge [17].

Transcriptomic studies of the *C* *. gigas* immune cells allowed the identification of a large set of immune related genes associated to the surviving capacity of oyster to Vibriosis in controlled conditions. The up regulation of genes related to immune response signaling, cellular processes and phagocytosis, but also antioxidant and anti-apoptotic reactions, has been identified in oysters able to survive virulent *Vibrio* infections [18]. In the same context, a hemocyte gene expression signature that predicted the oyster capacity to survive a Vibrio infection has been defined [19]. However, little information exists on the immune basis of oyster divergent selection based on surviving capacity to summer mortalities. This requires an increased knowledge of the differences on the immune response between oyster lines contrasted in terms of surviving capacity.

In the present study, we aimed to characterize sequence polymorphism and gene expression variability of several immune related genes on non-stimulated oysters from two selected lines which showed contrasted survival capacity in field conditions [8]. For this, we analyzed oysters from both H and L selected lines, which did not experience any abnormal mortality, and compared the gene expression of 42 immune related genes between the two lines. Results showed that the variability of gene expression and the sequence polymorphism acting on particular genes could enable the discrimination between H and L oyster lines. Additionally, the L oyster line seems to display higher levels of sequence polymorphism compared to the H line, suggested by melting temperature and sequencing analyses, and evidence of positive selection in some AMPs. We also found that the variability on *Cg*-Defs and *Cg-*Prp antimicrobials gene expression might be generated by gene copy number variations. Overall, this data gives new insights in the relationship between oyster immunity and divergent phenotypes, and discusses the potential implication of antimicrobial diversity in oyster survival to summer mortalities.

## Materials and Methods

### Oyster lines

*Crassostrea* *gigas* oysters used in this study come from two oyster lines selected by divergent selection for High (H) or Low (L) survival to summer mortality phenomenon in 2001 [6,7]. Furthermore, the lines were the same as those tested in [8], which still exhibited contrasted survival in field condition in 2009. Oyster lines from one full-sib family were reproduced within lines for six generations without any further round of selection (for further details on larval and spat culture methods and selection criteria, see [5–8,20]). In order to keep the “L” line as susceptible to mortality, oysters were always protected from risk factors throughout the generations, because breeding the survivors of the “L” line would have undeniably increased their resistance due to the high heritability of survival in juvenile *C* *. gigas* [5-7]. The sixth generation of both oyster lines were spawned on February 2009 at the IFREMER hatchery in La Tremblade (Charente Maritime, France), and as soon as the juveniles were caught on a 2 mm mesh screen, they were transferred to the IFREMER nursery located in Bouin (Vendée, France) on April 2009. During the nursery step, 5000 oysters per line were placed in a sieve of 500 mm of diameter and both lines were hold in the same tank. Raw seawater was hold in a pond, which was refilled once per day during the high tide, and then distributed in the nursery. The seawater was enriched with a cultured microalgae *Skeletonema* *costatum* to favor the oyster growth and a flow-through upwelling system alimented each sieve. Oysters were cleansed twice a week using seawater, and juveniles *C* *. gigas* within the same line were gently mixed to avoid environmental effect on growth. The complete experimental procedure was optimized to avoid contact of oysters with mortalities occurring in the field or any other stress, in order to obtain non-stimulated oysters from both lines and reared in the same conditions. For this, oysters were disposed at the IFREMER-Bouin nursery, in the same tank, with recirculating, filtered and ultraviolet light-disinfected water from the local environment. Cultured microalgae ( *S* *. costatum* ) were daily added as food source. Oysters between 7 and 10 months old were transferred in less than 24 hours to the IFREMER facilities located at Palaeovas (Languedoc Roussillon, France) in September 2009 and December 2009.Oysters remained one week in the same tank with recirculating, filtered and ultraviolet light-disinfected seawater from the local environment for acclimation before sampling. Cultured microalgae ( *S* *. costatum* ) were daily added as food source. The sampling was performed as follows: Non-stimulated oysters were removed from their shells and the whole soft body was immediately plunged in liquid nitrogen. Then, oysters were individually pulverized with a Mixer Mill MM 400 (Retsch) under liquid nitrogen conditions and frozen oyster powder was placed in cryogenic tubes to be stored at -80°C until use.

### RNA extraction and cDNA synthesis

RNA extraction was performed following the TRIzol Reagent manual according to manufacturer’s instructions (Invitrogen). Frozen oyster powder (20 mg) was homogenized in 1 ml of Trizol by vortexing between 1-2 h at 4°C. Prior to extraction, insoluble materials were removed by centrifugation at 12000xg for 10min at 4°C. Next, RNA samples were treated with 5 units of DNase I (Invitrogen) to eliminate DNA contamination, followed by heat inactivation (10 min at 65°C). Finally, RNA was precipitated with 100% isopropyl alcohol and 3 M Na-acetate, washed with 75% cold ethanol and dissolved in 50 µl of RNase-free water. Quantification and integrity of total RNA was checked using a *NanoDrop* spectrophotometer (Thermo, Fisher Scientific) and 1.5% agarose gel electrophoresis, respectively. Total RNA (3 µg) was reverse transcribed in 20 µl using the Moloney Murine Leukemia Virus Reverse Transcriptase (MMLV-RT) according to manufacturer’s instructions (Invitrogen).

### Genomic DNA extraction

For the genomic DNA (gDNA) extractions, 20 mg of frozen oyster powder was homogenized in 500 µl of lysis buffer (100 mM NaCl, 10 mM Tris-HCl, 25 mM EDTA, 0.5% SDS and 0.1 mg/ml proteinase K, pH 8) for 4 h at 50°C, followed by phenol/chloroform extraction and precipitation with 100% ethanol for 2 h at -80°C. gDNA was spooled with a pipette tip and washed in tubes containing 75% ethanol, vacuum-dried, dissolved in DNase-free water and treated with RNAse (Invitrogen) 50 µg/ml for 30 min at 37°C. A second precipitation was performed with 100% isopropyl alcohol and 3 M Na-acetate and the gDNA pellet was resuspended in DNase-free water. Quantification and integrity of gDNA was checked using a *NanoDrop* spectrophotometer (*NanoDrop* Thermo, Fisher Scientific) and 0.8% agarose gel electrophoresis, respectively.

### Gene expression analysis by quantitative PCR

qPCR assays were carried out on the Light-Cycler 480 System (Roche). The 5 µl-volume reaction consisted of 1X Light-Cycler 480 master mix, 0.5 µM of each primer and 1 µl of cDNA diluted at 1/8 in sterile ultra-pure water. qPCR assays were performed in triplicate, and primer pair efficiencies (E) were calculated by five serial dilutions of pooled cDNA ranging from 1/2 to 1/64 in sterile ultra-pure water, in duplicate with each primer pair. Primer pair efficiencies were calculated from the given slopes in LightCycler software according to the equation: E = 10[–1/slope]. List of primers used to amplify the 42 immune related genes and the three reference genes are presented in Table **S1**. qPCR assays were submitted to an initial denaturation step of 15 min at 95°C followed by an amplification of the target cDNA (35 cycles of denaturation at 95°C for 10 s, annealing at 57°C for 20 s and extension time at 72°C for 25 s) and fluorescence detection. Relative expression was calculated using the method described by Pfaffl [21], using the mean of the measured threshold cycle (Cq) values of three constitutively expressed genes (*Cg-EF1* [GenBank AB122066], *Cg-RPL40* [GenBank FP004478] and *Cg-RPS6* [GenBank HS119070]) to normalize the measured Cq values of target genes.

### Polymorphism detection: melting curves analysis and sequencing

At the end of the qPCR reaction and after an initial 10 s denaturation step at 95°C, a melting curve was obtained from a start temperature of 65°C to a final temperature of 95°C, with an increase of 0.06 °C/s with continuous fluorescence detection. When variation of 0.5°C on the melting temperature was detected between samples, a classical PCR was performed with a different pair of primers which amplified the complete open reading frame (ORF) using Taq DNA polymerase according to manufacturer’s protocol (Promega). List of primers are presented in Table **S2**. Then, 1X Syber Green was added to the PCR product and melting temperatures of amplicons were obtained by applying melting curve program on the Light-Cycler 480 System (Roche). Amplicons from classical PCR showing differences of melting temperature as observed from qPCR were cloned using TOPO TA cloning kit with the pCR 2.1 TOPO vector (Invitrogen), according to manufacturer’s protocol. Plasmid DNA (pDNA) from positive clones was purified using the WizardR SV miniprep DNA purification kit (Promega). Inserts were sequenced using a Big Dye Terminator sequencing kit on a DNA sequencer model ABI Prism 3130XL (Applied Biosystems) using universal M13 forward primer.

### Gene copy number estimation by quantitative PCR

Gene copy number of *Cg-Defs* and *Cg-Prp* was estimated by qPCR. For each gene, specific pairs of primers were designed from a conserved region on one single exon (listed in Table **S2**). For gene quantification a standard curve was generated using equimolar amounts of pCR 2.1 TOPO pDNA (plasmid DNA) containing the inserts of *Cg-Defs* and *Cg-Prp* were used in 40 ng of herring sperm DNA. PCR efficiency was calculated as described above, but from serial dilutions ranging from 10^3^ to 10^9^ copies of pDNA per reaction. qPCR assays were then performed in duplicate with 20 and 40 ng of gDNA per reaction. Reaction was performed as described above, with an initial denaturation step of 15 min at 95°C, 40 cycles of denaturation at 95°C for 10 s, annealing at 60°C for 20 s and extension time at 72°C for 20 s, and fluorescence detection followed by melting curve acquisition. The data were analyzed using Light Cycler 480 software version 1.5.0.39 and the 2nd derivative max algorithm. Gene copy number was calculated by absolute quantification. Standard curves for gDNA and pDNA of each gene were constructed from the mean Cq values using linear regression, from which slope and correlation coefficients were calculated. Using an estimation of the *C* *. gigas* genome size of about 824 Mb [22], the number of genome equivalents represented in 20 ng of gDNA was then calculated. Quantification was finally achieved by plotting the Cq values on the standard curve obtained with the serial dilutions of pDNA.

### Sequence and statistical analysis

The multiple alignments were generated using the BioEdit program [23] and translated sequences were obtained using ExPASy Translate tool [24]. Hierarchical clustering was determined using Multi Experiment Viewer [25]. Prediction of signal peptide was performed with the SignalP program [26]. The amino acids under selective pressure were detected by the ratio of the rate of non-synonymous substitutions (dN) to the rate of synonymous substitutions (dS) for each codon, calculated with Selecton web server [27], based on M8 evolutionary model which allows for positive selection. Statistical significance of results (*p*<0.05) was assessed using the likelihood ratio test (LRT) which compares the log likelihood of M8 model to the log likelihood of M8a alternative model, which allows for negative and neutral selection. Statistical analyses were performed using STATISTICA software version 7.1 (StatSoft) using Shapiro-Wilk test to test the normality of the data (significant value: *p*<0.05); Mann-Whitney U test to compare melting temperature between oyster lines at the level of oyster groups (significant value: *p*<0.05) and for expression analysis, Student’s t-test to compare melting temperature between oyster lines at individual level (significant value: *p*<0.05), Fisher test to compare variance of melting temperature within oyster lines at individual level (significant value: *p*<0.05), and Spearman’s rank correlation coefficient to compare expression level and gene copy number (significant value: *p*<0.05 or *p*<0.1).

## Results

### Differential expression levels of immune related genes are observed according oysters lines

**Figure 1:**
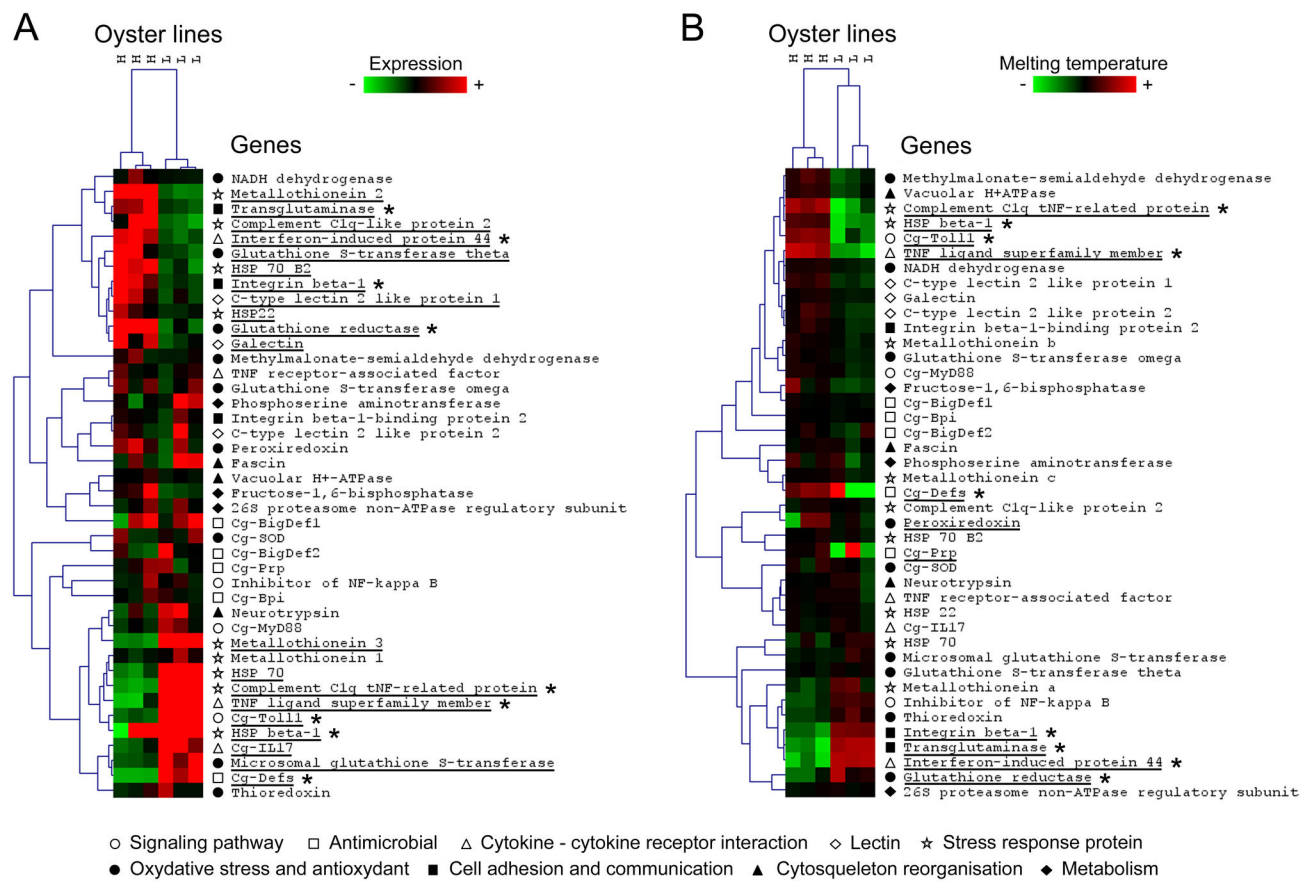
Gene expression and sequence polymorphism of 42 immune related genes in two oyster lines. Selected genes belonging to nine functional categories are listed below the figure and represented as symbols in front of each gene name. **A**) Hierarchical clustering of the relative expression levels of 42 immune related genes in non-stimulated oysters of H and L line (three groups of ten oysters per line). Each cell in the matrix corresponds to the expression level of one gene in one sample. The intensity of the color from green to red indicates the magnitude of differential expression (see color scale at the bottom of the image). Relative expressions were calculated according the 2^−ΔΔCq^ method [21]. The dendrogram at the top of the figure indicate relationship among samples; while the dendrogram at the right of the figure indicate relationship among the relative expression levels of selected genes. Hierarchical clustering was constructed with Multiple ArrayViewer software using average linkage clustering with Spearman Rank Correlation as the default distance metric. Significant differences of relative expressions between oyster lines were determined by the Mann-Whitney U test and genes with significant variation are underlined (*p*<0.05). (**B**) Hierarchical clustering of the melting temperatures of qPCR amplicons of 42 selected genes in non-stimulated oysters of H and L line (three groups of ten oysters per line). Melting temperature of each sample is represented as the deviation from the mean of melting temperatures of all samples for each gene. Each cell in the matrix corresponds to the deviation from the mean of melting temperature of one gene in one sample. The intensity of the color from green to red indicates the magnitude of the deviation of melting temperature from the mean of each gene (see color scale at the bottom of the image). The dendrogram at the top of the figure indicate relationship among samples; while the dendrogram at the right of the figure indicate relationship among variation of melting temperatures of selected genes. Hierarchical clustering was constructed with Multiple Array Viewer software using average linkage clustering with Pearson Correlation as the default distance metric. Genes who present variation equal or superior to 0.5°C between samples and/or a significant differences of melting temperature between oyster lines (Mann-Whitney U test, *p*<0.05) are underlined. Asterisks (*) indicate genes who present a significant differential of expression together with a variation of melting temperature.

We first investigated the differences on the gene expression of immune related genes between two oyster lines selected for the high (H) and low (L) resistance to summer mortalities. For this, we measured by qPCR the level of expression of 42 immune-related genes at constitutive level, i.e. in non-stimulated oysters (three groups of ten oysters per line). The list of selected genes is presented in Table **S1** and includes genes from nine functional categories. Results showed a high variability on the immune gene expression levels, which allowed the discrimination of the two oyster lines (Figure **1A**). From the 42 analyzed genes, 20 were differentially expressed between lines of non-stimulated oysters, including 11 genes mainly expressed in H line and 9 genes mainly expressed in L line (Mann-Whitney U test, *p*<0.05). Moreover, some differentially expressed genes could be associated with functional categories. For example, two of the three analyzed genes from lectin and from cell adhesion-communication categories appeared to be associated to H oyster line with a higher expression levels compared to L line. On the opposite, two functional categories display genes associated to L line, but with a lower number of differentially expressed genes per category. For instance, only one gene (*Cg-Toll*) from the signaling pathways category is differentially expressed with a higher level of expression in L line, as well as in the antimicrobials category, where only one differentially expressed gene (*Cg-Defs*) is detected with a higher level of expression in L line. Others functional categories cannot be associated to a specific oyster line because they display a comparable number of highly expressed genes in both lines, such as stress response proteins and cytokine-cytokine receptor interaction. Some others categories did not display a significant number of differentially expressed genes between oyster lines. We also observed a high variability on the gene expression of *Cg-Prp*, *Cg-BigDef1* and 2, *inhibitor of NF-kappa-B cactus*, *C-type lectin 2 like* protein *1*, *fascin*, and *phosphoserine amino transferase* 1 within oyster lines, which lack differential expression. Others genes such as *Cg-Bpi*, *Vacuolar H+ATPase*, and *NADH dehydrogenase* display a low variability of gene expression within lines.

**Figure 2:**
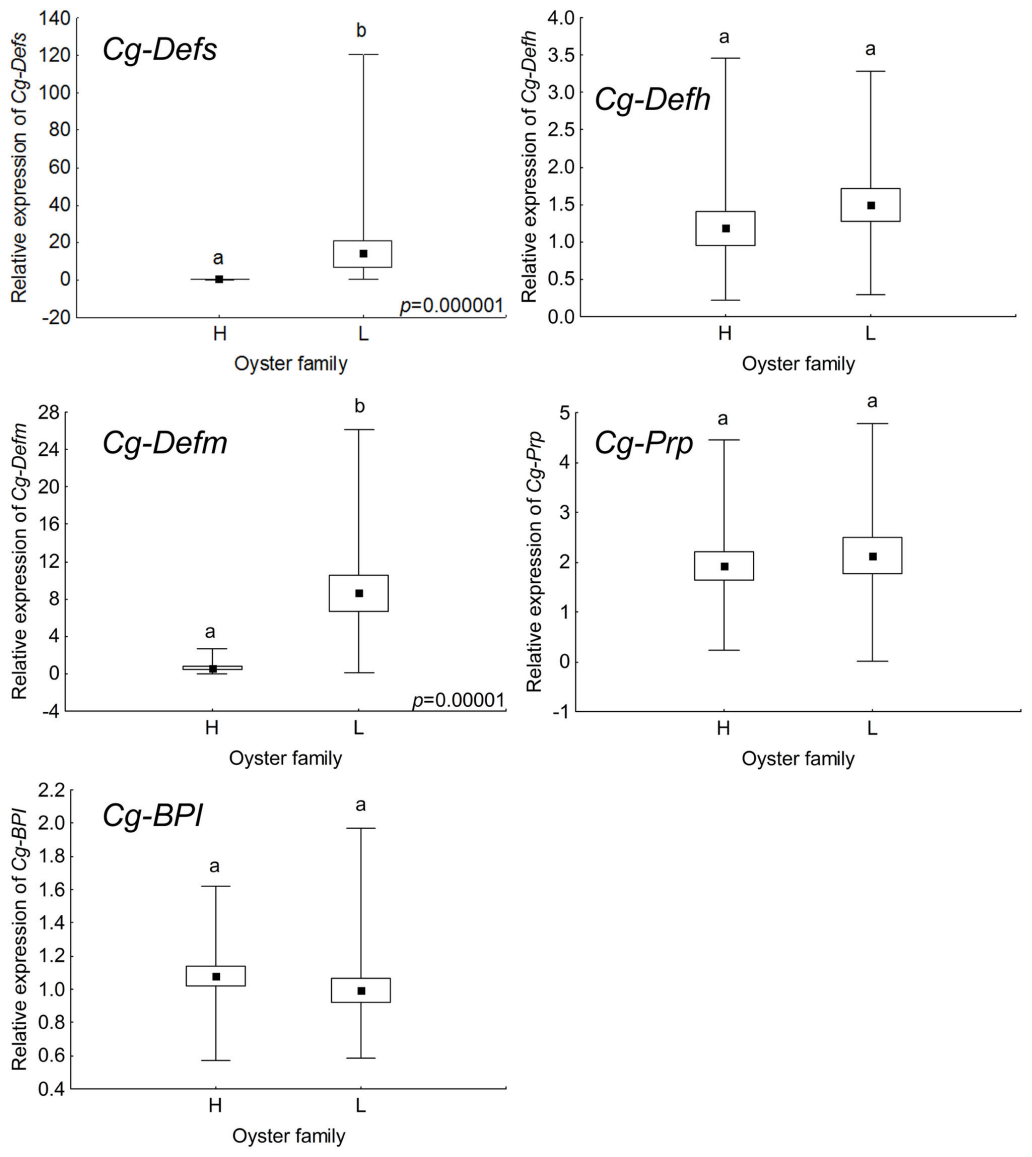
Gene expression of five antimicrobials in individual oysters from H and L lines. Relative expression of antimicrobial peptides (*Cg-Defs*, *Cg-Defh*, *Cg-Defm*, and *Cg-Prp*) and protein (*Cg-BPI*), were obtained from H and L selected oyster lines (19 oysters per line). Relative expression are expressed as box plot, where central point represents the mean, box represents ± standard error, and plot represents minimum and maximum value. Relative expression levels were obtained according to the 2^−ΔΔCq^ method [21]. Significant differences of relative expressions between oyster lines are indicated by different lowercase letters (different letters indicate significant difference, a or b) and were determined by the Mann-Whitney U test (*p*<0.05).

Because variability of expression could be related to only a few individuals or be a common feature among oysters, we then evaluated the expression of a limited number of immune-related genes on individual oysters. For this, we chose three antimicrobial families namely the defensins (*Cg-Defs*), the Proline Rich Peptides (*Cg-Prp*) and the Bactericidal-Permeability Increasing protein (*Cg-BPI*), which are representatives of the different expression profiles found from groups of animals (three groups of ten oysters per line). This means, differentially expressed between oyster lines, displaying high variability of expression among oyster lines, and displaying low variability. Expression of *Cg-Defs* genes was monitored using (i) universal primers to amplify all defensin variants, referred to as *Cg-Defs* and (ii) specific primers amplifying single variants, referred to as *Cg-Defhs* for hemocyte defensins and *Cg-Defm*, for mantle defensin. Results from individual analysis showed that the expression of both, overall *Cg-Defs* and *Cg-Defm* genes could enable the discrimination between the two oyster lines (Mann-Whitney U test, *p*<0.05) (Figure **2**). Interestingly, *Cg-Defs* and *Cg-Defm* showed a higher expression in the L line. On the opposite, *Cg-Defh* showed a high variability of expression among individuals and could not discriminate oyster lines. Expression of *Cg-Prp* and *Cg-BPI* also showed no significant difference between oyster lines. However, the expression of *Cg-Prp* was notably more diverse than that of *Cg-BPI*, and was even not detected for one individual of the L line.

### Differential patterns of sequence polymorphism on immune related genes are observed between oyster lines.

#### By melting temperature analyses

Sequence polymorphism was first evaluated by the analysis of melting temperature of amplicons generated by qPCR during the gene expression analyses of the 42 immune related genes (Figure **1B**). We included on this analysis three reference genes (Table **S1**). For the analysis, we considered that a shift on the melting temperature of 0.5°C between the amplicons of the same gene, from two different samples, could be indicative of polymorphism in the nucleotide sequence [28]. Results showed a variation on the melting temperature between amplicons from 11 genes, distributed in six functional categories (Figure **1B**). Only genes encoding lectins or involved in the cytoskeleton reorganization category did not display any variation on the melting temperature. We detected eight genes displaying significant variation on their melting temperatures between oyster lines by Mann-Whitney test (i.e. *C1q TNF-related protein* (Z=-1.99, *p*=0.046), *HSP* beta-*1* (Z=1.96, *p*=0.049), *TNF ligand super family member* (Z=1.99, *p*=0.049), *Transglutaminase* (Z=-1.99, *p*=0.046), and *Interferon-induced* protein *44* (Z=-1.99, *p*=0.046), *Cg-Toll* (Z=1.96, *p*=0.049), *glutathione* reductase (Z=-1.99, *p*=0.046), *integrin* beta-*1* (Z=1.96, *p*=0.049)). Among these genes, some of them display higher variability on their melting temperatures in one line compared to the other. *Cg-Toll* and *glutathione reductase* genes appear to present higher polymorphism in L line, while *integrin* beta-*1* appears to present higher polymorphism in H line. At the same time, the *peroxiredoxin* and the AMPs *Cg-Defs* and *Cg-Prp* genes display a variation on their melting temperatures of 2.4°C, 1.2°C and 0.6°C among samples, respectively, but with no significant difference among oyster lines. Interestingly, the high variation of their melting temperatures seems to be associated to one oyster line. Thereby, the AMPs *Cg-Defs* and *Cg-Prp* genes appear to display higher polymorphism in L line compared to H line according standard deviation (-0.22±1.34 vs. 0.22±0.04 and -0.05±0.62 *vs.* 0.05±0.05, respectively), while *peroxiredoxin* appears to display higher polymorphism in H line compared to L line (0.02±0.33 vs. -0.02±0.07, respectively).

**Figure 3:**
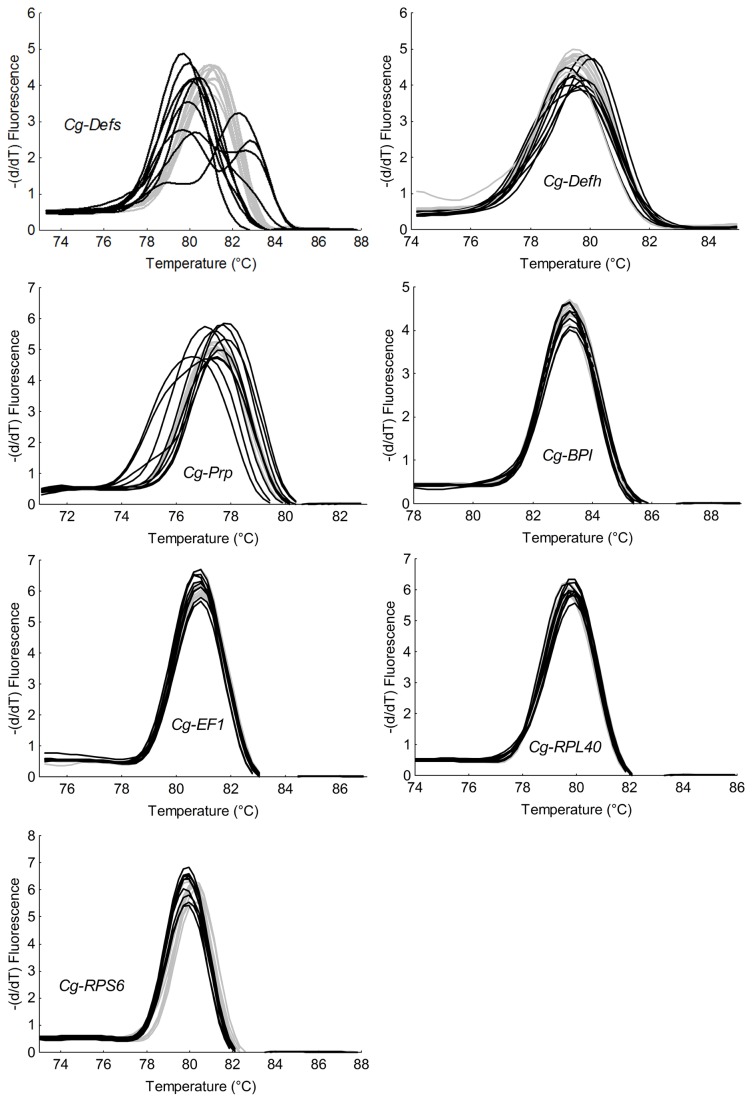
Melting temperature from transcript amplicons of four antimicrobial peptides and protein and three reference genes in individual oysters from H and L lines. Graphs represent melting curves of qPCR amplicons of three antimicrobial peptides (*Cg-Defs*, *Cg-Defh*, and *Cg-Prp*), one antimicrobial protein (*Cg-BPI*), and three constitutively expressed genes (*Cg-EF1*, *Cg-RPL40* and *Cg-RPS6*) from two selected oyster lines (ten oysters per line). H oyster line is represented in grey and L oyster line in black. The three antimicrobial peptides (*Cg-Defs*, *Cg-Defh*, and *Cg-Prp*) display a high variation on their melting temperatures is significantly associated with the L line (Fisher test, *p*<0.05) while other genes present same variations in each oyster lines (Fisher test, *p*>0.05).

In order to evaluate the potential sequence polymorphism on individual oysters, we focused on the three antimicrobials analyzed before (*Cg-Defs*, *Cg-Prp*, and *Cg-BPI*). As in the gene expression analyses, the variation of the melting temperature of *Cg-Defs* was monitored using (i) universal primers to amplify all defensin variants, referred as *Cg-Defs* and (ii) specific primers amplifying specific variants, referred as *Cg-Defhs* for hemocyte defensins and *Cg-Defm*, for mantle defensin. Due to the weak gene expression of *Cg-Defm*, the melting temperature of this gene was not considered in this analysis. Results showed for *Cg-Defs*, *Cg-Defh* and *Cg-Prp* amplicons a variation on their melting temperatures of 2.6°C, 0.9°C, and 1.1°C respectively, at the individual level (Figure **3**). The high variation on their melting temperatures is significantly associated with the L line, (Fisher test: *Cg-Defs* ratio F: 40.5, *p*=0.00002; *Cg-Defh* ratio F: 4.2E+10, *p*=2.02E-46; *Cg-Prp* ratio F: 15.3, *p*=0.0003), but this variability does not allow the discrimination between oyster lines (Student’s t-test, *p*>0.05). This analysis suggests the existence of a higher nucleotide polymorphism for these antimicrobials in L line compared to H line. In contrast, no variation on the melting temperatures for *Cg-BPI* or the three reference genes was found (Student’s t-test, *p*>0.05 and Fisher test, *p*>0.05), suggesting the absence of polymorphism on these genes (Figure **3**).

#### By ORF sequencing

**Figure 4:**
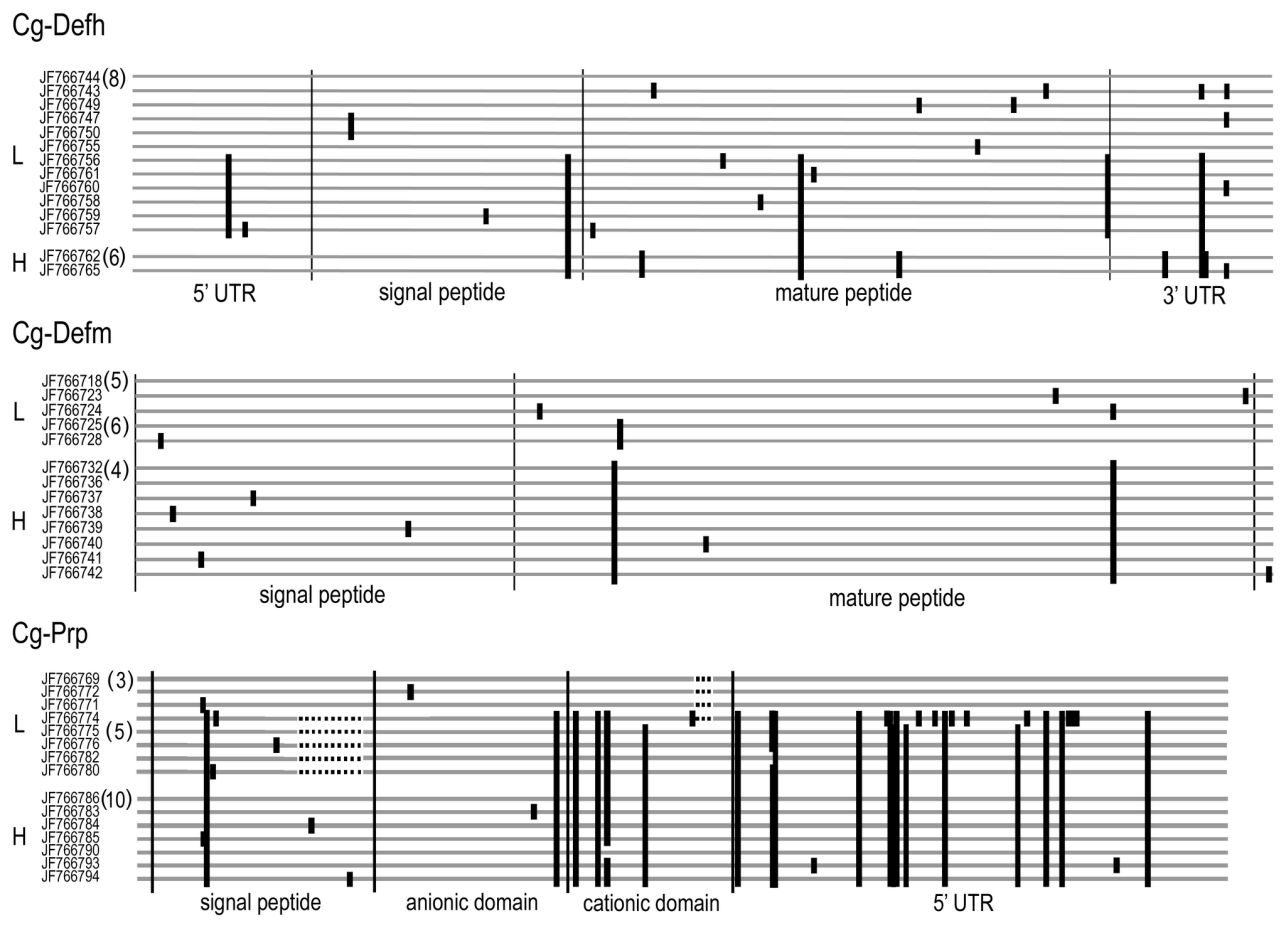
Schematic alignment of transcript sequences of three antimicrobial peptides, *Cg*-*Defh*, *Cg*-*Defm* and *Cg*-*Prp* from H and L oyster lines. Transcript sequences were obtained by PCR from whole oyster body RNA, from L lines (L) and H line (H) oysters. Numbers between parentheses at the left of sequences indicate the number of identical sequences found. Black bars indicate polymorphic sites compared to the first sequence.

To gain more evidence about the polymorphism predicted by the melting temperature analysis, we sequenced the entire ORF of the AMPs *Cg-Defh, Cg-Defm* and *Cg-Prp* amplified from three groups of ten oysters per line. A mean of 13 exploited sequences for each AMP per oyster line seems to confirm that *Cg-Defh*, *Cg-Defm* and *Cg-Prp* present a higher polymorphism in the L line compared to the H line (Figure **4**). Sequences are available at NCBI: *Cg-Defh*-L line (GenBank ID: JF766743-JF766761), *Cg-Defh*-H line (GenBank ID: JF766762- JF766768), *Cg-Defm-*L line (GenBank ID: JF766718-JF766731), *Cg-Defm*-H line (GenBank ID: JF766732-JF766742), *Cg-Prp*-L line (GenBank ID: JF766769-JF766782) and *Cg-Prp*-H line (GenBank ID: JF766783-JF766798).

We identified two major sequences for each *Cg-Defh* and *Cg-Defm* in the L line, while only one sequence of each variant was identified in the H line. Therefore, the *Cg-Defs* family seems to display a higher polymorphism in the L line. In addition, no common *Cg-Defs* sequences were found among oyster lines, as a result of 10 and three major polymorphic sites (mutation found in at least two sequences) for *Cg-Defh* and *Cg-Defm*, respectively.

For *Cg-Prp*, three groups of sequences differing in the length of the coding region were identified in the L line, while only one group was identified in the H line. Thus, *Cg-Prp* also appears to display a higher sequence polymorphism in the L line. The three lengths of sequences encode (i) a new variant of the original *Cg-Prp* previously published [13], showing a deletion of 21 nucleotides in signal peptide coding sequence; (ii) the shorter *Cg-Prp* produced by an indel of six nucleotides in the cationic domain coding sequence [14] and (iii) and a new shorter variant, presenting both deletions described above. The presence of 20 major polymorphic sites allows the discrimination of long and short variants, but not oyster lines. Finally, as also observed for *Cg-Defs*, no common *Cg-Prp* sequences were found among oyster lines due to the indel present in signal peptide. Remarkably, the only *Cg-Prp* variant detected in H line is the original long variant described before [13], and the new short variant found in L line is the shortest *Cg-Prp* sequence identified until now.

### Each oyster line displays a particular repertory of AMP variants and different selection pressures are observed between oyster lines AMPs

**Figure 5:**
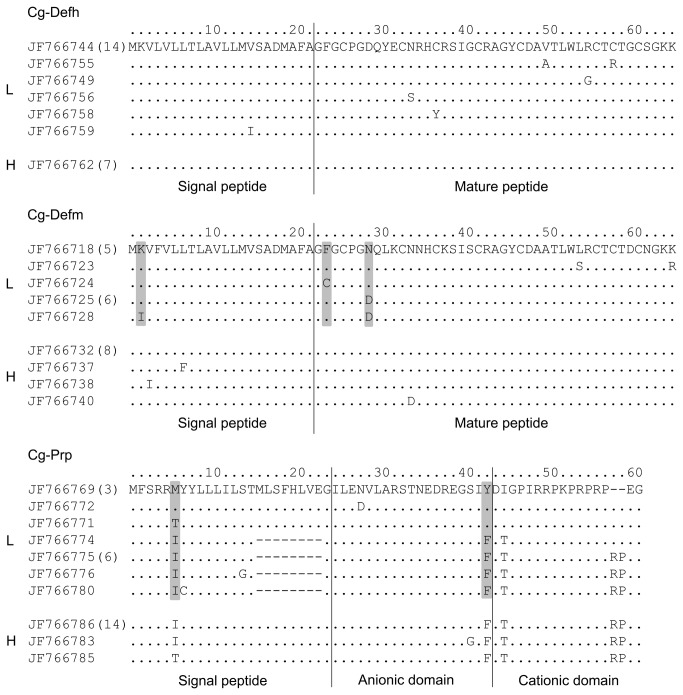
Alignment of deduced amino acid sequences of three antimicrobial peptides, *Cg*-Defh, *Cg*-Defm and *Cg*-Prp, from H and L oyster lines. Amino acid sequences were deduced from transcripts sequences obtained from L lines (L) and H line (H) oysters. Numbers between parentheses at the left of sequences indicate the number of identical sequences found. Black bars indicate substitution sites and dots show identical amino acids compared to the first sequence. Amino acids under positive selection are shown in gray (using the ratio of nonsynonymous to synonymous substitutions per codon).

The biological function of the AMPs resides mainly on their primary peptide structure [29]. Therefore, to evaluate the consequences of the nucleotide polymorphism at the amino acid level, we focused on the nonsynonymous mutations in the ORF of the AMP sequences. Particularly, we analyzed if the observed polymorphism have been shaped by positive selection pressure, as supporting evidence of higher diversity. The presence of sites under positive selection was calculated by the ratio of nonsynonymous to synonymous substitutions (dN/dS) per codon [27]. To assess the significance of the findings, we carried out a likelihood ratio test (LRT) and considered only coded amino acids showing significantly positive selection (*p*<0.05) (Figure **5**).

Despite the presence of several major polymorphic sites in *Cg-*Defh ORF, they were all synonymous and gave rise to the same encoded peptide in both lines (Figure **5**). Only single sequences from the L line showed nonsynonymous polymorphic sites (positions 15, 34, 37, 50, 55 and 58) but without evidence of positive selection. In contrast, three of the eight nonsynonymous mutations found in *Cg-*Defm sequences from L line seem to be under positive selection (positions 2, 24, and 29). Interestingly, the nonsynonymous mutation at position 29 produced a radical substitution in the mature peptide region (Asp/Asn). Likewise, two of the seven nonsynonymous mutations found in *Cg*-Prp sequences from the L line seem to be under positive selection (position 6 and 43). Interestingly, the nonsynonymous mutation at position 43 is located at the junction between the anionic and cationic domains of the mature peptide (Tyr/Phe). Nevertheless, the putative chymotrypsin cleavage site between domains remained unchanged [13]. In addition, two indels events were found in *Cg*-Prp sequences from the L line. The first indel produces a loss of eight amino acids in the signal peptide region of some sequences. The second indel produces the shorter variant lacking a Pro-Arg motif [14] (Figure **5**). The evidence of sites under positive selection pressures in *Cg-*Defm and *Cg*-Prp and the existence of the two indels in *Cg*-Prp sequences support the hypothesis of a higher polymorphism of *Cg-*Defm and *Cg*-Prp AMPs in L line compared to H line.

### Variability of AMP gene expression is correlated with the gene copy number and sequence polymorphism could be encoded in the oyster genome

To gain insight into the genetic bases of the high variability of gene expression observed for oyster AMPs, we studied the potential correlation between gene copy number and the gene expression levels. For this, 14 oyster individuals that had already been analyzed for gene expression were randomly selected for gene copy number estimation. Gene copy number and relative expression from whole oyster body were determined for the AMP families *Cg-Defs* and *Cg-Prp* by quantitative PCR. Additionally, melting curves from cDNA and genomic DNA (gDNA) were used for sequence polymorphism evaluation on transcript and genomic sequences, respectively.

**Figure 6:**
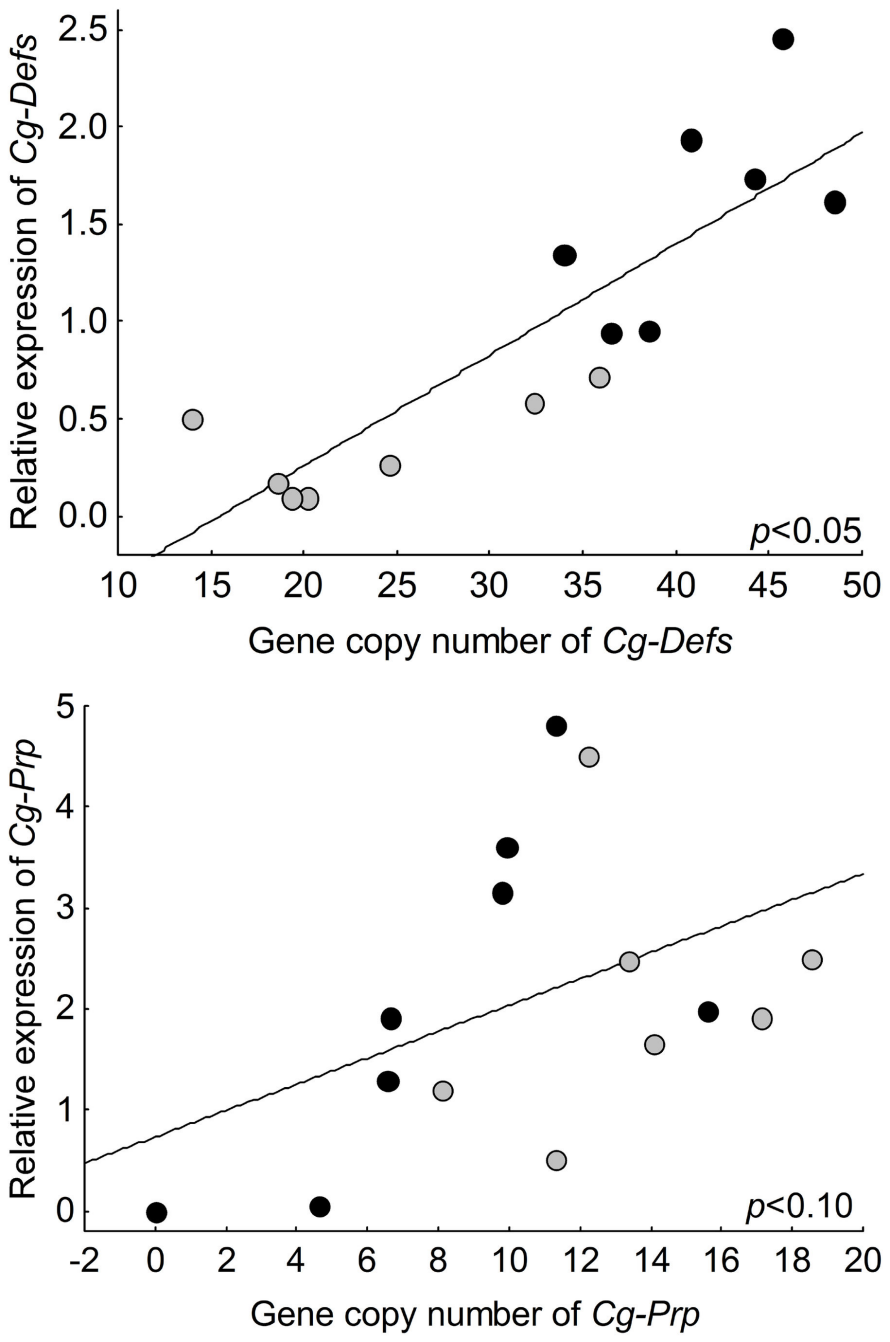
Correlation between basal gene expression and gene copy number for two antimicrobial peptides *Cg-Defs* and *Cg-Prp* from two oyster lines. Relative expression and gene copy number were estimated by qPCR (N=14 or 15) for (a) *Cg-Defs* and(b) *Cg-Prp*. Individuals from L oyster line are shown in black, individuals from H oyster line are shown in grey. Significant positive correlations between expression level and gene copy number (Spearman’s rank correlation coefficient) were detected for *Cg-Defs* (*p*<0.05) and *Cg-Prp* (*p*<0.1).

For *Cg-Defs*, we observed a significant positive correlation between the gene copy number and gene expression, with a variation of 14 to 48 gene copies among individuals (*p*<0.05). The L line displays a higher gene copy number and relative expression than the H line (Figure **6a**). Moreover, as predicted from cDNA melting curves analyses, results from gDNA showed higher variability of melting temperatures in L line compared to the H line (Fisher test: ratio F: 24.5, *p*=0.0004) which suggest higher polymorphism in the genomic *Cg-Defs* sequences in L line (Figure **S1**).

For *Cg-Prp*, we detected a lower but still significant positive correlation between the gene copy number and gene expression, with a variation ranging from the absence of detection (in one individual of L line) to 18 gene copies among individuals (0.10>*p*>0.05) (Figure **6b**). As observed for *Cg-Defs*, melting curve analyses from gDNA showed higher variability of melting temperatures in L line compared to the H line (Fisher test: ratio F: 2.4E+24, *p*=1.5E-84) which suggest higher polymorphism in the genomic *Cg-Prp* sequences in L line (Figure **S1**).

## Discussion

Our results showed a high variability on the gene expression and sequence polymorphism of certain immune related genes of *C* *. gigas* , which allow the discrimination between two oyster lines selected for High (H) or Low (L) capacity to survive summer mortalities. We analyzed the basal expression and sequence polymorphism of 42 immune related genes from non-stimulated oysters. The absence of prior stimulation of oysters was verified by the lack of differential expression of genes described as inducible by bacterial stimulation in *C* *. gigas* , such as the *inhibitor of NF-kappa-B* [30] and the AMPs *Cg-BigDef1* and *Cg-BigDef2* [17]. We identified 20 immune related genes showing a differential expression between oyster lines, and 11 displaying potential sequence polymorphism. Finally, we showed that basal gene expression level of AMP in individual oysters could be driven by gene copy number and sequence polymorphism could be encoded in the genome.

Differentially expressed genes are found in almost all functional categories and certain categories can be associated to a specific oyster line. For example, the lectin, and cell adhesion and communication categories display genes with higher expression in the H line compared to the L line. Within the lectin category, we identified a *C-type lectin* which has been described as a mediator of various immune responses after pathogen recognition [31,32]. We also identified a *galectin*, which can bind to glycans on the surface of potentially pathogenic microbes, acting as a recognition factor [33]. Therefore, the high level of expression of these two lectins may be beneficial for pathogen recognition in the H line. Within the cell adhesion and communication category, we identified *transglutaminase* and β*-integrin* genes. Transglutaminase was shown to be a coagulation factor [34,35] which can regulate the expression of several immune related genes [36]. The differential expression of transglutaminase and lectin related genes have already been described between oyster lines with different tolerances to heat stress [37]. Thus, it is tempting to speculate that they could improve disease resistance in the H oyster line. Interestingly, the oyster pathogen *V. splendidus* LGP32 subverts β-integrins to invade oyster hemocytes [38]. Therefore, we could not correlate the higher expression of this gene with the survival capacity of the H oyster line. However, we cannot rule out the hypothesis that over-expression of β-integrin gene could be involved in other functions.

Others functional categories cannot be associated to a specific oyster line because they display, (i) a comparable number of highly expressed genes in both lines, or (ii) a low number of differentially expressed genes. The first case is observed in stress response proteins from multigenic families (*C1q domain-containing like proteins*, *metallothioneins*, and *heat shock proteins*) for which variants have been identified between oyster lines showing different susceptibility to summer mortalities or heat stress [9,37,39]. Thus, the expression of certain variants of stress response proteins could be correlated to the surviving capacity of oysters. On the second case, for example, only the AMP family *Cg-Defs* from the antimicrobials category appeared differentially expressed with a higher level of expression in L line. Strikingly, a previous analysis showed contradictory results with a higher level of *Cg-Defs* gene expression in tissues (gills and muscle) of the resistant oyster line during a mortality event in the field [9]. However, this difference could be related to the abundant migration of circulating hemocytes expressing this AMP to sites of infection during the mortality event [15]. Others functional categories such as cytoskeleton reorganization and metabolism lacked differentially expressed genes among the limited number of genes analyzed here.

In contrast to gene expression, the variability on melting temperatures, nucleotide sequences, and the finding of sites under positive selection pressures support the hypothesis that the polymorphism of certain immune related genes could allow the discrimination between oyster lines. High polymorphism in *C* *. gigas* genome [40,41] and in transcript sequences [15,42,43] have been described before and appear to be a characteristic of this species.

Sequence polymorphism between lines was observed for eight genes associated to signaling pathways, oxidative stress and antioxidants, cytokine and interacting proteins, stress response proteins, and cell adhesion and communication functional categories. Interestingly, polymorphism on these genes has been associated to differential susceptibility to autoimmune diseases in human populations. Tumor necrosis factor super family (TNFSF) polymorphism has been related to the susceptibility to multiple sclerosis [44,45] and cancer [46,47]. Polymorphism on interferon-induced proteins and C1q domain-containing proteins were associated to susceptibility to psoriasis and diabetes [48,49] and to systemic lupus [50], respectively.

Concerning the sequence polymorphism within lines, we detected a high polymorphism in two AMP families (*Cg-Defs* and *Cg-Prp*), in *Cg-Toll* and in *glutathione reductase* genes in oysters from the L line. Remarkably, these AMP families display variants with differential *in vitro* antimicrobial activities [15]. Different variants for the *Cg-Toll* receptor were described in green mud crab and they have been related to resistance to *Vibrio* infection [51]. Similarly, rare mutations in the *glutathione reductase* gene caused hereditary glutathione reductase deficiency in humans [52]. On the other hand, we detected a high sequence polymorphism in peroxiredoxin and in integrin β-1 genes from the H line oysters. Peroxiredoxin is an antioxidant enzyme and several variants have been identified in *C* *. gigas* . It has been suggested that they might be under selective effect of environmental stress [53]. Therefore, the sequence polymorphism identified in our study could have consequences on the differential susceptibility of the H and L oyster lines to summer mortalities and needs further investigation. It is now of prime importance to evaluate the biological activities of these variants to understand the molecular basis of the differential resistance of oyster lines.

Results also showed that AMPs could enable the discrimination between H and L oyster lines, by variability of gene expression and/or sequence polymorphism from the analysis on individual oysters. Particularly, the discrimination of oyster lines by sequence polymorphism was highlighted by the higher variability of melting temperatures in L line, which is linked to different nucleotide sequences and to the presence of amino acids under positive selection pressure. In addition, the high variability of gene expression of the AMP families *Cg-Defs* and *Cg-Prp* is correlated to gene copy number polymorphism. Sequence polymorphism of *Cg-Defm* and *Cg-Prp* could be coded in the oyster genome and oyster AMPs are represented as multigenic families with an amazing number of genes, as described elsewhere [14]. Although we confirmed the existence of the major polymorphic sites in the public databases for these AMPs and we identified new variants, we cannot exclude the presence of other polymorphic sites that only greater sequencing efforts can determine.

Strikingly, *Cg-Defs* and *Cg-Prp* display higher variability of gene expression and/or sequence polymorphism in the L line. Indeed, compared to the L line, H oysters showed a lower number of genes and a lower gene expression for *Cg-Defs*, and lower sequence polymorphism for *Cg-Defm* and *Cg-Prp*. One explanation could be that the selection process of oyster lines has decreased the genetic diversity of the H line, selecting only one expressed variant with better performance. Higher numbers of genes and/or higher expression of AMPs could be detrimental to the host, supported by the evidence that a higher number of AMP gene copies is not necessarily related with an enhanced immune protection. Copy number variations have been associated to human diseases [54], and when acting on immune genes they could possibly contribute to immune differences between individuals [55,56]. Therefore, future work must be oriented towards the comprehension of this correlation that may contribute to successful immune response in oyster.

It would be of great interest to determine if the decrease in the genetic diversity of the H line is related to antimicrobial peptides with enhanced activity. For instance, the second indel found in the *Cg*-Prp sequences from the L line produced the deletion of one Pro-Arg motif in the cationic active region of the peptide. Since Pro-Arg motifs are involved in the antimicrobial activity of proline-rich AMPs [57,58], this mutation may have a functional importance. Indeed, the shorter variant found in L line is less active *in vitro* than the long variant found in H line [15]. The Asp to Asn amino acid mutation found in fixed variant of *Cg-*Defm in the H line, which is the same amino acid under positive selection in the L line, could increase the affinity of this peptide to negatively charged bacterial membranes and improve their antimicrobial activity [59]. Accordingly, the role of particular variants of AMPs in the susceptibility or resistance of oyster lines to oyster mortalities in the field remains to be elucidated.

The H oyster line seems to have fixed lower expression levels and/or sequence diversity of AMPs. This may reflect differential surviving capacities to summer oyster mortalities, as proposed for specific repertoires of AMPs in natural populations of northern leopard frogs [60], or for the differential of expression of two chicken β-defensins between resistant and sensible inbred lines to *Salmonella* infection [61].

In conclusion, our results suggest that the variability of gene expression and the sequence polymorphism acting on particular innate immune genes could enable the discrimination between H and L oyster lines, and that this variability might be generated by gene copy number variations. Therefore, study of the significance of the correlation between gene expression and copy number polymorphism, as well as functional studies on AMPs variants should be of prime importance for understanding the traits which govern resistance of oysters to summer mortalities.

## Supporting Information

Figure S1 **Melting temperatures from genomic DNA of two antimicrobial peptides, *Cg-Defs* and *Cg-Prp* from individual oysters of H and L lines.** Graphs represent melting curves of qPCR amplicons of *Cg-Defs* and *Cg-Prp* from individuals of two selected oyster lines (four oysters in replicate per line). Individuals from L oyster line are shown in black; individuals from H oyster line are shown in grey. *Cg-Defs* and *Cg-Prp* showed higher variability of melting temperatures in L line compared to H line (Fisher test, *p*<0.05).
(TIF)

Table S1 **List of primers used to amplify 42 immune related genes and three reference genes from *C* *. gigas* whole body RNA by qPCR.**
(XLSX)

Table S2 **List of primers used for PCR amplification from cDNA and gene copy number estimation of antimicrobial peptides, *Cg-Defs* and *Cg-Prp* from *C* *. gigas* .**
(XLSX)
