# Supplementary figures and images for: Sequence Polymorphism and Expression Variability of Crassostrea gigas Immune Related Genes Discriminate Two Oyster Lines Contrasted in Term of Resistance to Summer Mortalities
Source: PLoS One. 2013 Sep 26;8(9):e75900. doi: 10.1371/journal.pone.0075900 (PMC3784401; doi:10.1371/journal.pone.0075900)

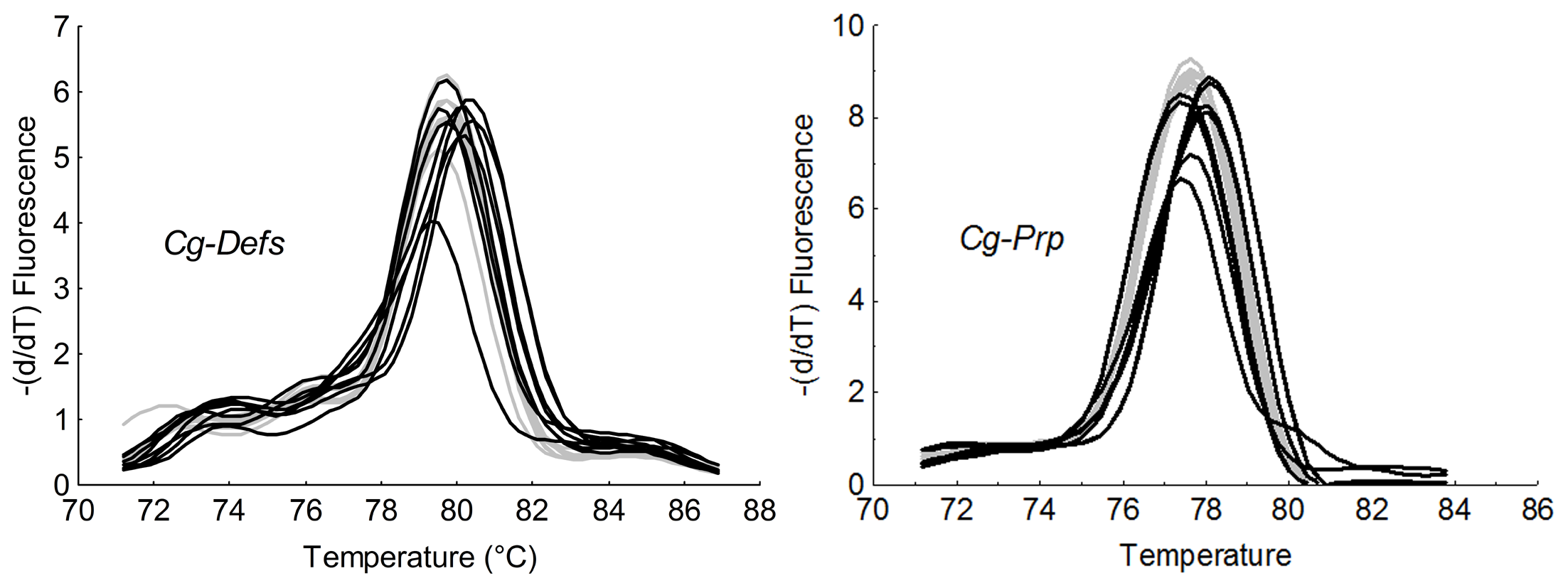

Supplement: Figure S1 — Melting temperatures from genomic DNA of two antimicrobial peptides, Cg-Defs and Cg-Prp from individual oysters of H and L lines. Graphs represent melting curves of qPCR amplicons of Cg-Defs and Cg-Prp from individuals of two selected oyster lines (four oysters in replicate per line). Individuals from L oyster line are shown in black; individuals from H oyster line are shown in grey. Cg-Defs and Cg-Prp showed higher variability of melting temperatures in L line compared to H line (Fisher test, p<0.05). (TIF) [file pone.0075900.s001.tif]
